# Cerebellar pilocytic astrocytoma in a patient with autism spectrum disorder and psychotic symptoms: a case report

**DOI:** 10.1186/s12888-025-07706-2

**Published:** 2025-12-25

**Authors:** Kanji Itami, Keitaro Kimoto, Yuki Takahashi, Yuichi Onishi, Katsunaka Mikami, Kenji Yamamoto

**Affiliations:** 1 Department of Psychiatry, Tokai University School of Medicine, 143 Shimokasuya, Isehara, Kanagawa 259-1193 Japan; 2 Aiko Hospital, 2 Chome-7-1 Matsue, Atsugi, Kanagawa 243-0005 Japan

**Keywords:** Cerebellar pilocytic astrocytoma, Autism spectrum disorder, Psychosis, Schizophrenia, Brain tumor, Hallucination

## Abstract

**Background:**

Psychiatric symptoms, including psychotic manifestations such as hallucinations and delusions, are common in patients with brain tumors, typically associated with tumors in the frontal and temporal lobes. Psychotic symptoms are rarely linked to cerebellar tumors. However, recent evidence suggests that the cerebellum is involved in higher-order functions like cognition, emotional regulation, and social behavior. This report describes the case of a patient with autism spectrum disorder (ASD) who presented with a unique combination of a cerebellar tumor and psychotic symptoms, achieved complete remission of psychotic symptoms without antipsychotic treatment, and was followed up for three years.

**Case presentation:**

A 16-year-old male with longstanding ASD presented with auditory hallucinations, delusional beliefs, and thought disorder. Since childhood, he displayed features such as poor eye contact, hypersensitivity to sounds, solitary play, restricted interests, and behavioral rigidity. At age 13, he began experiencing fluctuating psychotic symptoms, which were later diagnosed as schizophrenia-like psychotic features alongside his ASD. Brain magnetic resonance imaging (MRI) revealed a 3-cm cerebellar mass, which was identified as a pilocytic astrocytoma upon surgical resection. Notably, the patient’s psychotic symptoms completely resolved after surgery, without the use of antipsychotic medication, and have not recurred during a 3-year follow-up, while his core characteristics of ASD remained unchanged.

**Conclusions:**

This case suggests that cerebellar pathology may contribute to the development of psychotic symptoms, supporting the hypothesis that cerebellar dysfunction can lead to schizophrenia-like features. It also highlights the potential link between cerebellar dysfunction and ASD. Given the slow-growing nature of pilocytic astrocytomas, the case highlights the importance of considering cerebellar pathology in the differential diagnosis of psychosis, with implications for understanding disorders like ASD.

## Background

Psychiatric symptoms are common in patients with brain tumors. Van der Meer et al. reported that the prevalence of depression and anxiety in patients with glioma was 16–41% and 24–41%, respectively [1]. Psychotic symptoms such as hallucinations and delusions occur in approximately 22% of patients, most often in those with tumors affecting the cerebral cortex, particularly the frontal and temporal lobes [2].

Psychiatric symptoms in brain tumor patients arise from a complex interplay of factors—not only tumor location but also tumor size, associated edema, growth rate, disruption of surrounding neural networks, epileptic activity, endocrine disturbances, treatment effects (surgery, radiotherapy, steroids, antiepileptic drugs), and psychosocial stressors [2]. However, substantial evidence also supports the relationship between tumor location and psychiatric manifestations. Frontal lobe lesions are associated with depressive symptoms, anxiety, apathy, personality changes, disinhibition, and occasionally longstanding psychosis [3]. Temporal lobe tumors frequently present with anxiety, affective instability, irritability, fear episodes, hallucinations, delusions, dissociation, and déjà vu phenomena [4]. Parietal lobe involvement often results in apraxia and acalculia, whereas occipital lobe lesions are linked to visual field defects, visual agnosia, and visual hallucinations such as distortions of light or shape [4]. Cerebellar tumors, although less likely to produce psychosis, are associated with the classical “cerebellar cognitive affective syndrome,” characterized by emotional blunting or dysregulation, executive dysfunction, and language disturbances [5].

Recent studies suggest a link between cerebellar dysfunction and autism spectrum disorder (ASD) or attention-deficit/hyperactivity disorder (ADHD) [6]. Previous case reports have described ASD co-occurring with cerebellar damage, and a hypothesis has been proposed that pilocytic astrocytoma, a slow-growing central nervous system (CNS) tumor typically found in childhood, may influence the development of ASD-related characteristics in early childhood [7].

Here, we report the case of a patient with cerebellar pilocytic astrocytoma who exhibited both ASD and psychotic symptoms. The present case is unique in several respects. The patient had a formal diagnosis of ASD, exhibited schizophrenia-like psychotic symptoms, and was found to have a cerebellar pilocytic astrocytoma. Notably, his psychotic symptoms resolved completely following tumor resection without antipsychotic treatment and did not recur over a 3-year follow-up period. No clear changes in ASD characteristics were observed before or after tumor resection. Such a constellation of ASD, cerebellar pathology, and fully reversible psychosis has rarely been reported and offers important insights into the role of the cerebellum in both psychosis and neurodevelopmental disorders.

## Case presentation

### Case:

A 16-year-old male high school student.

### Chief Complaint:

Hearing voices.

### Developmental history

The patient was born full-term via normal vaginal delivery with normal birth weight. There were no delays in motor or language development. At one year of age, he exhibited poor eye contact and hypersensitivity to certain sounds. He did not show interest in other children and preferred solitary play. While playing with toy trains, he was unable to stop midway, and when taken outside to see real trains, he refused to return home, showing difficulty with transitions. He exhibited strong fixations, such as insisting on following specific self-imposed rules and always taking the same route.

Even after starting elementary school, he continued to prefer solitary play and often had temper tantrums when things did not go as he wished. In summary, he showed persistent qualitative impairments in reciprocal social interaction, communication, and restricted interests, with a tendency toward rigidity, indicating impaired imaginative function.

After graduating from elementary school, he took a junior high school entrance exam and began boarding at an integrated junior and senior high school.

### Present illness

In the first year of junior high school (year X-4), he began experiencing auditory hallucinations, such as hearing voices talking to him or giving commands. He did not consult anyone about these symptoms and tried to cope alone. The symptoms were intermittent and disappeared at times. During his third year of junior high school (year X-1), the hallucinations temporarily subsided but returned intermittently. In his first year of high school (year X, month Y), he frequently visited the school infirmary, complaining of feeling unwell. After disclosing his auditory hallucinations and suicidal thoughts to the school nurse, he was referred to our hospital and visited for the first time on year X, month Y, day Z.

### Past medical history

No significant past medical history.

### Family history and home environment

The patient is an only child and lives with both parents. His father has a history of depression.

### Psychiatric and neurological findings

The patient was alert and oriented, with no disturbances in consciousness or memory. He reported experiencing auditory hallucinations, saying, “Sometimes I hear someone calling out to me.” He also exhibited paranoid delusions, such as “Someone is giving off signs that they want to kill me.” Thought disorder was evident in messages he sent to friends, such as “The immortal sweet roll. I was floating in the air. Next week I will go to Chen Guanyu.” There were no signs of disturbances in self-identity.

He lacked insight into his condition, stating, “I’m not ill, and I’m not having any problems.” No neurological abnormalities were observed.

### Laboratory and imaging findings

**Fig. 1:**
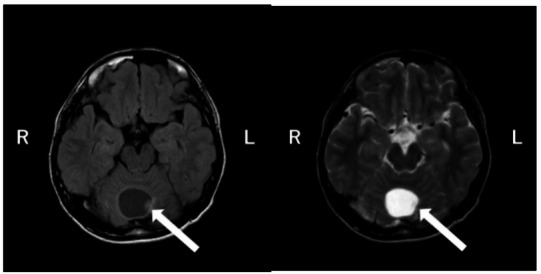
T1- and T2-weighted MRI images. T1- and T2-weighted brain MRI images show a well-circumscribed mass, approximately 3 cm in size, located in the cerebellar vermis. No other remarkable abnormalities were observed in the brain parenchyma or sulci

1. Wechsler Adult Intelligence Scale - Fourth Edition (WAIS-IV): Full-Scale Intelligence Quotient (IQ) 111; Verbal Comprehension 113; Perceptual Reasoning 107; Working Memory 119; Processing Speed 93.
2. Electroencephalogram (EEG): Background rhythm consisted of 10 Hz alpha waves. No paroxysmal abnormalities were observed.
3. Brain magnetic resonance imaging (MRI): A cerebellar tumor approximately 3 cm in diameter was identified (Fig. 1).

### Clinical diagnoses

The patient was evaluated by two board-certified psychiatrists, one with more than 5 years and the other with more than 15 years of clinical experience. A detailed developmental history was obtained from both the patient and his parents, along with clinical interviews and school information. Although no structured diagnostic instruments were administered, ASD was diagnosed according to DSM-5 criteria, which include: (1) deficits in social-emotional reciprocity, nonverbal communication used for social interaction, and the ability to develop, maintain, and understand relationships; (2) insistence on sameness, inflexible adherence to routines, and restricted interests; and (3) hypersensitivity to sensory stimuli. This clinical information was considered sufficient to meet the diagnostic threshold for ASD.

Schizophrenia was included in the differential diagnosis given the presence of psychotic symptoms. Although the psychiatric presentation met DSM-5 criteria for schizophrenia, the diagnosis was deferred because the cerebellar tumor could reasonably account for the psychiatric manifestations. Final diagnostic determination was postponed pending observation of the patient’s clinical course following treatment.

### Course of treatment

Regarding the cerebellar tumor, the attending psychiatrist consulted with a neurosurgeon, and surgical resection was deemed appropriate. Concerning the psychotic symptoms, a decision was made to observe the clinical course without initiating antipsychotic medication, in accordance with the preferences of the patient and his family.

**Fig. 2:**
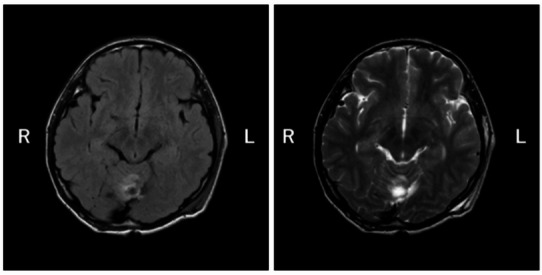
Postoperative T1- and T2-weighted MRI images. Postoperative T1- and T2-weighted brain MRI confirm the resection of the cerebellar vermis mass

In month Y + 2 of year X, a surgical resection of the tumor was performed (Fig. 2). Histopathological examination confirmed pilocytic astrocytoma. Following tumor resection, the patient’s psychotic symptoms—including auditory hallucinations, paranoid delusions, and thought disorder—resolved completely within a few days. No clear changes in core ASD characteristics were observed before or after surgery. Although core traits such as a preference for solitary activities and strong adherence to routines persisted, he adjusted to high school life, and his academic performance remained good.

No recurrence of psychotic symptoms was observed during the subsequent 3-year follow-up period, and the patient remained free of hallucinations, delusional ideation, or disorganized thinking. Notably, he did not receive antipsychotic medication at any point. In contrast, ASD-related characteristics remained unchanged. He continued to prefer solitary activities, adhere to fixed routines, and display mild pragmatic communication difficulties, with no significant exacerbation or improvement after surgery. Despite these ASD features, he was able to return to school, maintain necessary interactions with peers and teachers, and showed no overt behavioral problems or significant emotional instability. He completed high school and advanced to university, where he continues to attend classes regularly and, despite certain challenges, manages to adapt to campus life.

## Discussion and conclusions

We reported a case of cerebellar pilocytic astrocytoma in a patient with ASD who presented with psychotic symptoms. A notable feature was that although the patient initially sought psychiatric care for psychosis, further evaluation revealed both a cerebellar tumor and long-standing core characteristics of ASD. The psychotic symptoms resolved completely following surgical resection without the use of psychotropic medications, and there has been no recurrence for more than three years. This rapid and sustained remission strongly suggests that the cerebellar tumor played a central role in the emergence of psychotic symptoms.

Pilocytic astrocytoma is the most common CNS tumor in children, accounting for 15–25% of all pediatric CNS tumors and 25–35% of posterior fossa tumors. Its incidence peaks at 6–8 years of age, with no sex differences. As a WHO Grade I tumor, gross total resection is considered the standard first-line treatment [8].

Psychosis associated with pilocytic astrocytoma is rare, with most reports describing peduncular hallucinations. Some cases involve cerebellar tumors compressing the brainstem, including the midbrain, resulting in peduncular hallucinations [9], whereas others report hallucinations persisting in the setting of stable midbrain tumors after radiotherapy [10]. In contrast, the psychotic features in our patient—paranoid delusions, dialogic auditory hallucinations, and thought disorder—were more consistent with schizophrenia than with peduncular hallucinations.

Accumulating evidence links cerebellar dysfunction to schizophrenia-like psychotic symptoms through disruptions in the cortico–cerebellar–thalamic–cortical circuit (CCTCC), a pathway implicated in cognitive dysfunction in schizophrenia [11]. Auditory verbal hallucinations have also been associated with reduced gray matter volume in specific cerebellar regions [12] and with abnormal cerebellar functional connectivity [13]. Furthermore, cerebellar lesions, whether due to stroke [14] or tumors [15], have been associated with the onset of delusional ideation [15, 16]. These findings suggest that the mass effect of the cerebellar pilocytic astrocytoma in our case may have contributed to the development of schizophrenia-like psychotic symptoms.

The present case may also be interpreted within the framework of Schmahmann syndrome, or cerebellar cognitive affective syndrome (CCAS), which includes disturbances of executive function, affect regulation, language, and visuospatial processing following cerebellar injury [17]. Similarly, the concept of cognitive dysmetria, proposed by Schmahmann and Andreasen, describes disrupted coordination of mental processes within the CCTCC [18]. These models underscore the cerebellum’s integrative role in higher-order cognitive and affective regulation, extending beyond motor control. Thus, cerebellar pathology in this patient may have contributed to both psychotic symptoms and disturbances in social–cognitive functioning.

The psychiatric assessment prompted by psychosis also led to the diagnosis of ASD. Although individuals with ASD can exhibit transient psychotic-like symptoms [16], the patient’s rapid and complete remission of psychosis following tumor removal suggests that the psychotic symptoms were unlikely to be solely attributable to ASD. Previous reports have described comorbid ASD and cerebellar pilocytic astrocytoma [7]. Given the tumor’s slow-growing nature and typical onset in early childhood, some authors have speculated that congenital or early developmental factors may link pilocytic astrocytoma to the neurodevelopmental features of ASD. However, in the present case, no clear changes in core ASD characteristics were observed before or after tumor resection. Thus, although a possible influence of cerebellar dysfunction on neurodevelopment cannot be entirely excluded, a causal relationship between the tumor and the development of ASD remains unconfirmed.

## Data Availability

Data sharing is not applicable to this article as no datasets were generated or analyzed during the current study.

## Abbreviations

ASD: Autism Spectrum Disorder
ADHD: Attention-Deficit/Hyperactivity Disorder
CCAS: Cerebellar Cognitive Affective Syndrome
CNS: Central Nervous System
DSM-5: Diagnostic and Statistical Manual of Mental Disorders, Fifth Edition
MRI: Magnetic Resonance Imaging
WAIS-IV: Wechsler Adult Intelligence Scale - Fourth Edition
EEG: Electroencephalogram
IQ: Intelligence Quotient
WHO: World Health Organization
GMV: Gray Matter Volume
CCTCC: Cortico-Cerebellar-Thalamic-Cortical Circuit
AVH: Auditory Verbal Hallucinations
